# Supplementary material for: RSL24D1 sustains steady-state ribosome biogenesis and pluripotency translational programs in embryonic stem cells
Source: Nat Commun. 2023 Jan 23;14:356. doi: 10.1038/s41467-023-36037-7 (PMC9870888; doi:10.1038/s41467-023-36037-7)
Supplement: Supplementary file 1 — Supplementary Information [file 41467_2023_36037_MOESM1_ESM.pdf]

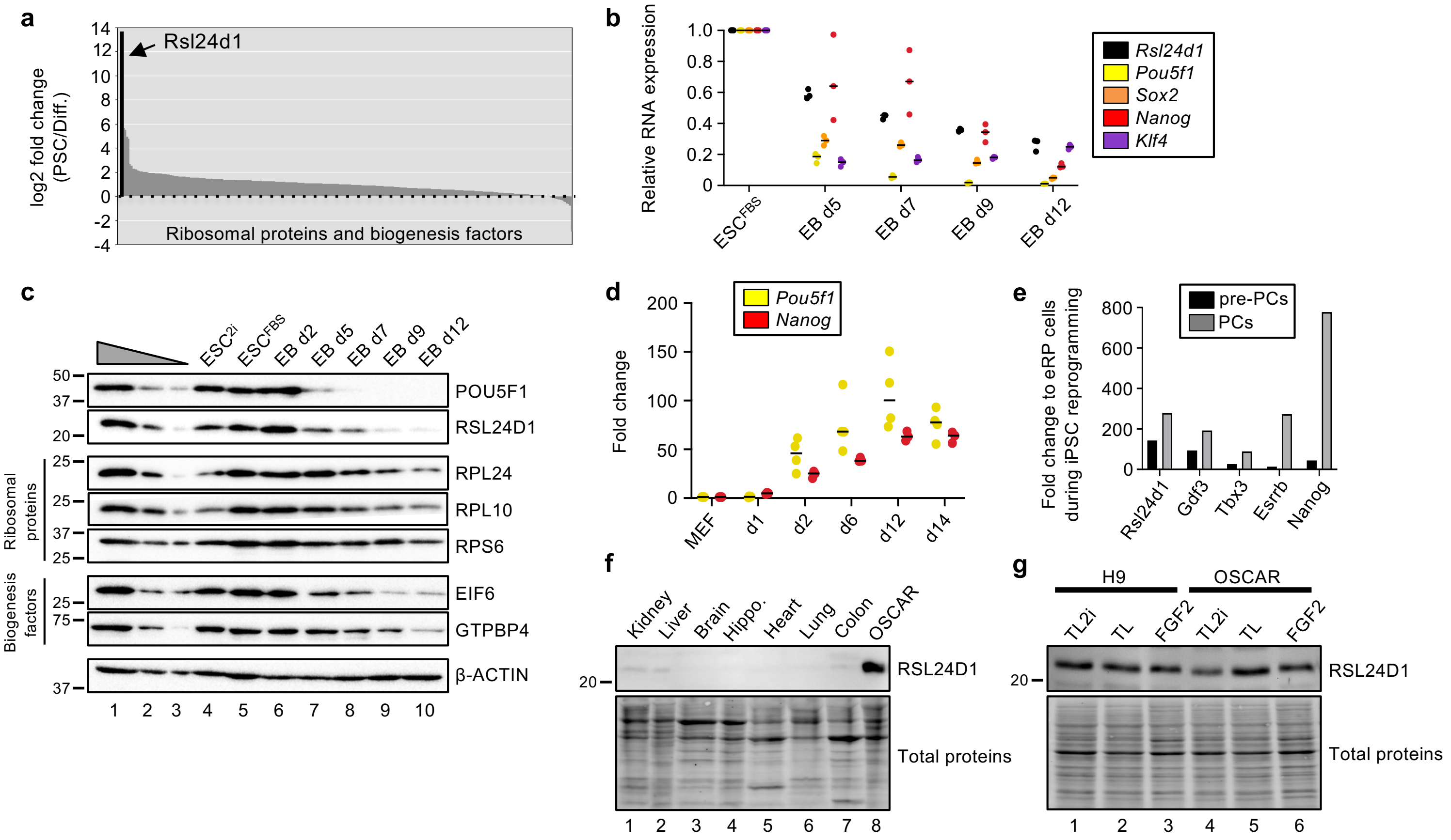

Supplemental Figure 1. RSL24D1 expression pattern in murine and human PSCs. **a** RNA-seq profiling of expression changes for 303 mRNAs encoding ribosomal proteins and ribosome biogenesis factors between mouse pluripotent and differentiated cells detailed in Supplementary Data 1. Expression changes are shown as log2 ratios of the average expression in PSCs (n= 7 cell lines) over differentiated cells (n= 6 cell lines), and ranked from the most enriched mRNAs in PSCs (left) to the most enriched in differentiated samples (right). **b** Quantitative PCR analysis of the expression of *Rsl24d1*, *Pou5f1*, *Sox2*, *Nanog* and *Klf4* mRNAs during a kinetic of CGR8 differentiation as embryoid bodies (EBs) at days 0 (ESC^{FBS}), 5, 7, 9 and 12 (n=3). Transcript levels are normalized to the β-Actin and Psmd9 mRNAs. Data are presented as mean values +/- SEM. **c** Representative immunoblots of RSL24D1, POU5F1, RPL24, RPL10, RPS6, EIF6 and NOG1 in total extracts from ground state (ESC^{2i}) and naïve pluripotent CGR8 (ESC^{FBS}) as well as EBs at 2, 5, 7, 9 and 12 days of differentiation (n=3). Lanes 1 to 3 correspond to serial dilutions of ESC^{FBS} extracts (1:1, 1:3 and 1:9, respectively). β-ACTIN is shown as a loading control. **d** Quantitative PCR analysis of the kinetics of expression of endogenous *Pou5f1* (n=4) and *Nanog* (n=3) mRNAs during the course of reprogramming of MEFs derived from doxycycline-inducible Col1a1-tetO-OKMS mice into iPSCs. Transcript levels are indicated relative to MEFs (day 0), at days 1, 2, 6, 12 and 14 post doxycycline induction, and are normalized to the *Tbp* and *Psmd9* mRNAs. Data are presented as mean values +/- SEM. **e** Single-cell RNA-seq gene expression changes in cells undergoing somatic reprogramming (pre-PCs) and chimera-competent pluripotent cells (PCs) compared to cells with early reprogramming potential (eRP) [30]. **f** Representative immunoblot of RSL24D1 in total extracts from a panel of human adult tissues and from pluripotent human OSCAR ESCs cultured in the presence of ectopic FGF2 (n=2). TCE labeled proteins (Total proteins) are used as loading control. **g** Representative immunoblot of RSL24D1 in total extracts from H9 and OSCAR human ESC lines maintained in naïve-like conditions (TL2i), in primed conditions with LIF + 4'OHT supplemented media (TL) supporting STAT3 overexpression or in presence of FGF2 [31] (n=3).

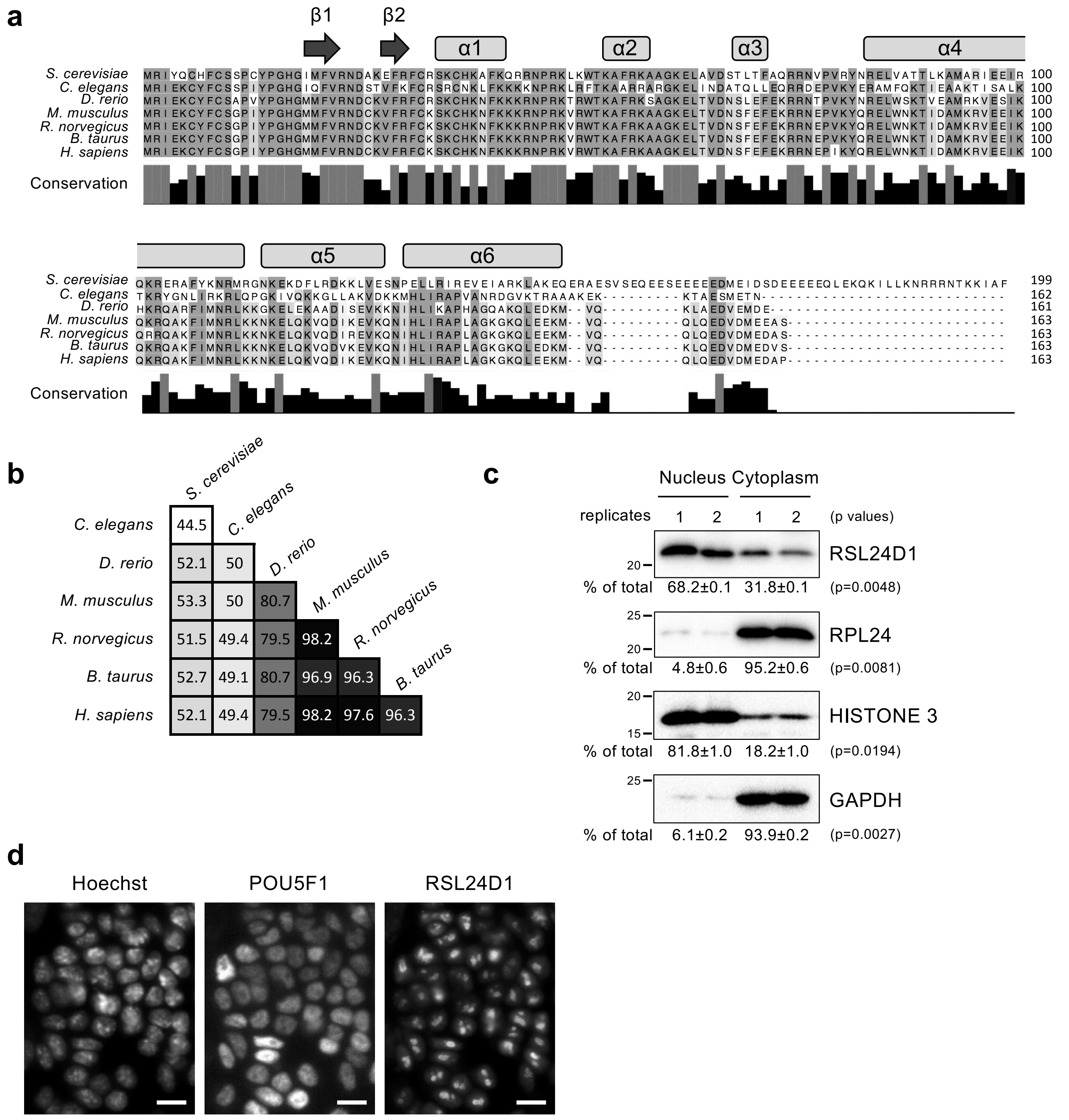

Supplemental Figure 2. RSL24D1 sequence and structure are conserved across evolution. a Multiple protein alignments of yeast Rlp24 with homologs from higher eukaryote species. Amino acid numbers are indicated on the right for each protein, and the conservation score to the yeast Rlp24 sequence for each position is calculated by the Jalview software and indicated below. The secondary structure predictions for the first 149 amino acids of the yeast Rlp24 protein are indicated above. β-sheets are represented by arrows and α-helixes by boxes. **b** Amino acid sequence identity calculated by paired-alignment between yeast Rlp24 and homologs from higher eukaryote species. RSL24D1 is highly conserved in higher eukaryotes from zebrafish to human. **c** Nucleo-cytoplasmic fractionation in two independent biological replicates of CGR8 cells cultured in naive conditions (ESC^{FBS}). Representative immunoblots of RSL24D1 and RPL24 are shown (n=2). HISTONE 3 and GAPDH are indicated as specific nuclear and cytoplasmic proteins, respectively. Quantifications of immunoblot signals in both nuclear and cytoplasmic fractions are estimated as percentage of total signals, defined by the sum of nuclear and cytoplasmic signal. Paired two tailed Student's *t* test. **d** Representative images of naïve CGR8 cells in ESC^{FBS} conditions stained with Hoechst, POU5F1 and RSL24D1 antibodies (20X objective) (n=3). The scale bar represents 10 µm.

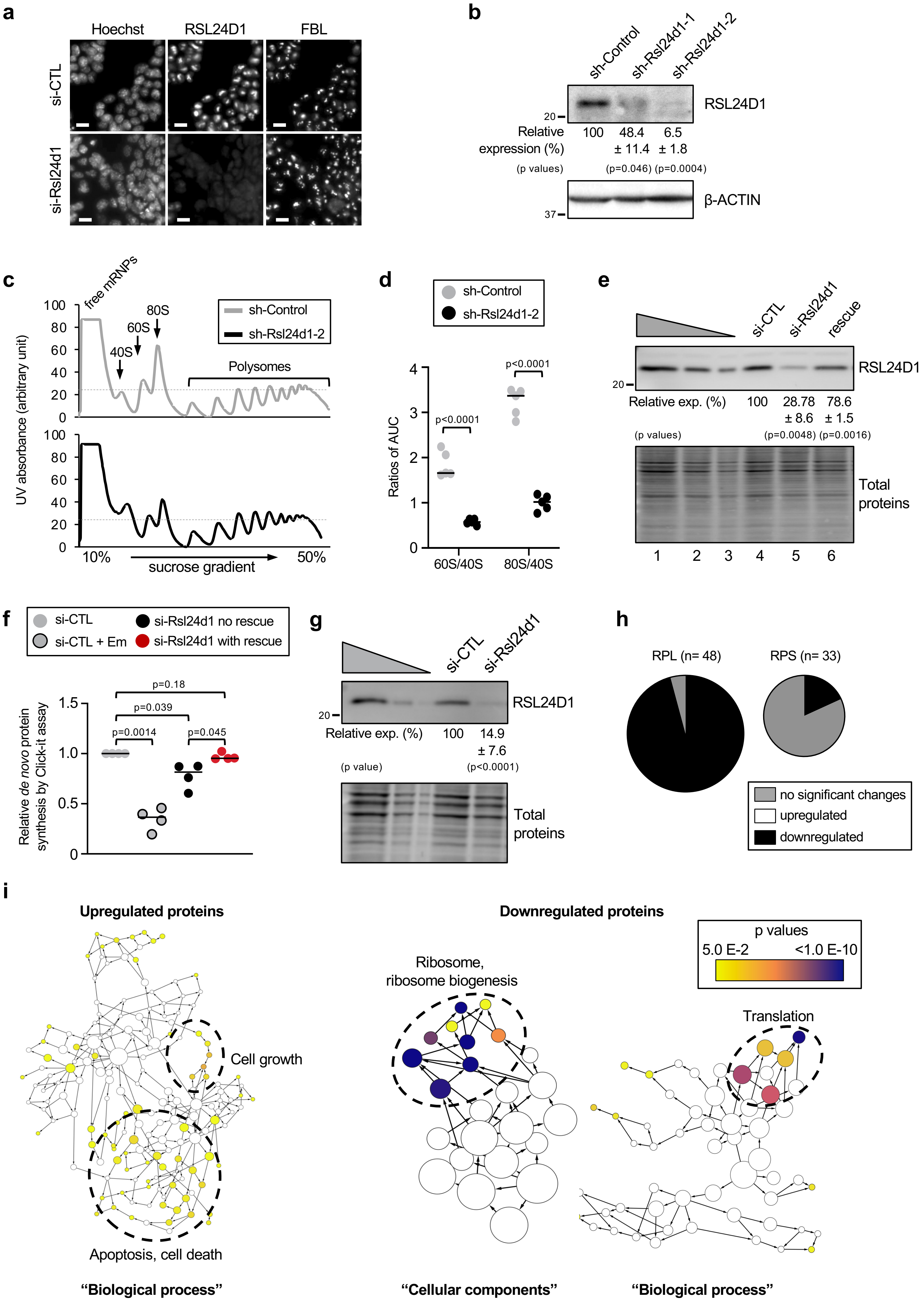
a
Hoechst
RSL24D1
FBL
si-CTL
si-Rsl24d1
b
sh-Control
sh-Rsl24d1-1
sh-Rsl24d1-2
RSL24D1
20
Relative expression (%)
100
48.4 ± 11.4
6.5 ± 1.8
(p values)
(p=0.046) (p=0.0004)
β-ACTIN
37
c
free mRNPs
40S
60S
80S
Polysomes
sh-Control
sh-Rsl24d1-2
UV absorbance (arbitrary unit)
10%
sucrose gradient
50%
d
sh-Control
sh-Rsl24d1-2
Ratios of AUC
p<0.0001
p<0.0001
60S/40S
80S/40S
e
si-CTL
si-Rsl24d1
rescue
RSL24D1
20
Relative exp. (%)
100
28.78 ± 8.6
78.6 ± 1.5
(p values)
(p=0.0048) (p=0.0016)
Total proteins
1
2
3
4
5
6
f
si-CTL
si-CTL + Em
si-Rsl24d1 no rescue
si-Rsl24d1 with rescue
Relative de novo protein synthesis by Click-it assay
p=0.18
p=0.039
p=0.0014
p=0.045
g
si-CTL
si-Rsl24d1
RSL24D1
20
Relative exp. (%)
100
14.9 ± 7.6
(p value)
(p<0.0001)
Total proteins
h
RPL (n= 48)
RPS (n= 33)
no significant changes
upregulated
downregulated
i
Upregulated proteins
Cell growth
Apoptosis, cell death
"Biological process"
Downregulated proteins
Ribosome, ribosome biogenesis
p values
5.0 E-2
<1.0 E-10
Translation
"Cellular components"
"Biological process"

Supplemental Figure 3. RSL24D1 depletion alters ribosome biogenesis and protein translation. a Representative images of si-CTL and si-Rsl24d1 treated naïve CGR8 cells stained with Hoechst and with anti-FBL and anti-RSL24D1 antibodies (20x objective) (n=3). The scale bars represent 10 µm. **b** Representative immunoblot of RSL24D1 (n=3) in total protein lysates from naïve CGR8 cells stably expressing control shRNAs or 2 independent Rsl24d1-targeting shRNAs. β-ACTIN is used as a loading control. RSL24D1 signals are normalized to β-ACTIN signals and expressed relative to the control shRNA expressing cells. Unpaired two tailed Student's *t* test. **c** Representative polysome profiling performed by centrifugation on sucrose gradient of cytoplasmic extracts from naive CGR8 cells infected with lentiviruses expressing control non-targeting shRNAs (grey) or shRNAs targeting Rsl24d1 (sh-Rsl24d1-2, black). 40S, 60S, 80S monosomes and polysomes are detected by UV-absorbance. **d** Dot plot indicating ratios of 60S/40S and 80S/40S absorbance peaks calculated by determining the area under the curve (AUC) for the 40S, 60S and 80S absorbance signals (n=5). Unpaired two tailed Student's *t* test. **e** Representative immunoblot of RSL24D1 expression in si-CTL, si-Rsl24d1 and si-Rsl24d1 with rescue cells (n=3). Lanes 1 to 3 correspond to serial dilutions of si-CTL (1:1, 1:3 and 1:9, respectively). TCE labeled proteins are used for normalization (Total Proteins). Quantifications of RSL24D1 signals normalized to total proteins and relative to the si-CTL condition (Relative exp. (%)) are indicated. Paired two tailed Student's *t* test. **f** Dot blot representing the *de novo* translation activity in CGR8 cells treated with si-CTL or with si-Rsl24d1, in the presence or absence of doxycyline. Emetine (Em.), a translation inhibitor, was used as a positive control of alterations of *de novo* protein synthesis. Protein synthesis was quantified by Click-it^{TM} AHA protein assay and analyzed by high-content screening microscopy (n=4). Multiple paired *t* test. **g** Representative immunoblot of RSL24D1 in total protein lysates from si-CTL and si-Rsl24d1 treated ESC^{FBS} CGR8 cells prior to mass-spectrometry analysis. Quantifications of RSL24D1 signals normalized to total proteins and relative to the si-CTL condition are indicated (n=3). Two tailed Student's *t* test. **h** Diagram representing the relative proportions of RPL (n=48) and RPS (n=33) affected by the siRNA mediated depletion of RSL24D1. Changes in protein levels detected by mass spectrometry are indicated by a colour code. The corresponding data are available in Supplementary Data 2. **i** Gene ontology (GO) enrichment analysis of biological process and cellular component terms significantly associated with proteins differentially expressed in si-Rsl24d1 treated CGR8 cells. The left and right panels represent hierarchical trees of the most enriched terms for up- or downregulated proteins in RSL24D1-depleted cells, respectively. The size of the nodes represents the numbers of genes associated to each GO term, and the corresponding p values defined by a hypergeometric test with Benjamini and Hochberg false discovery rate corrections are indicated by colour codes. The most represented GO term categories are indicated. The corresponding data are available in Supplementary Data 3.

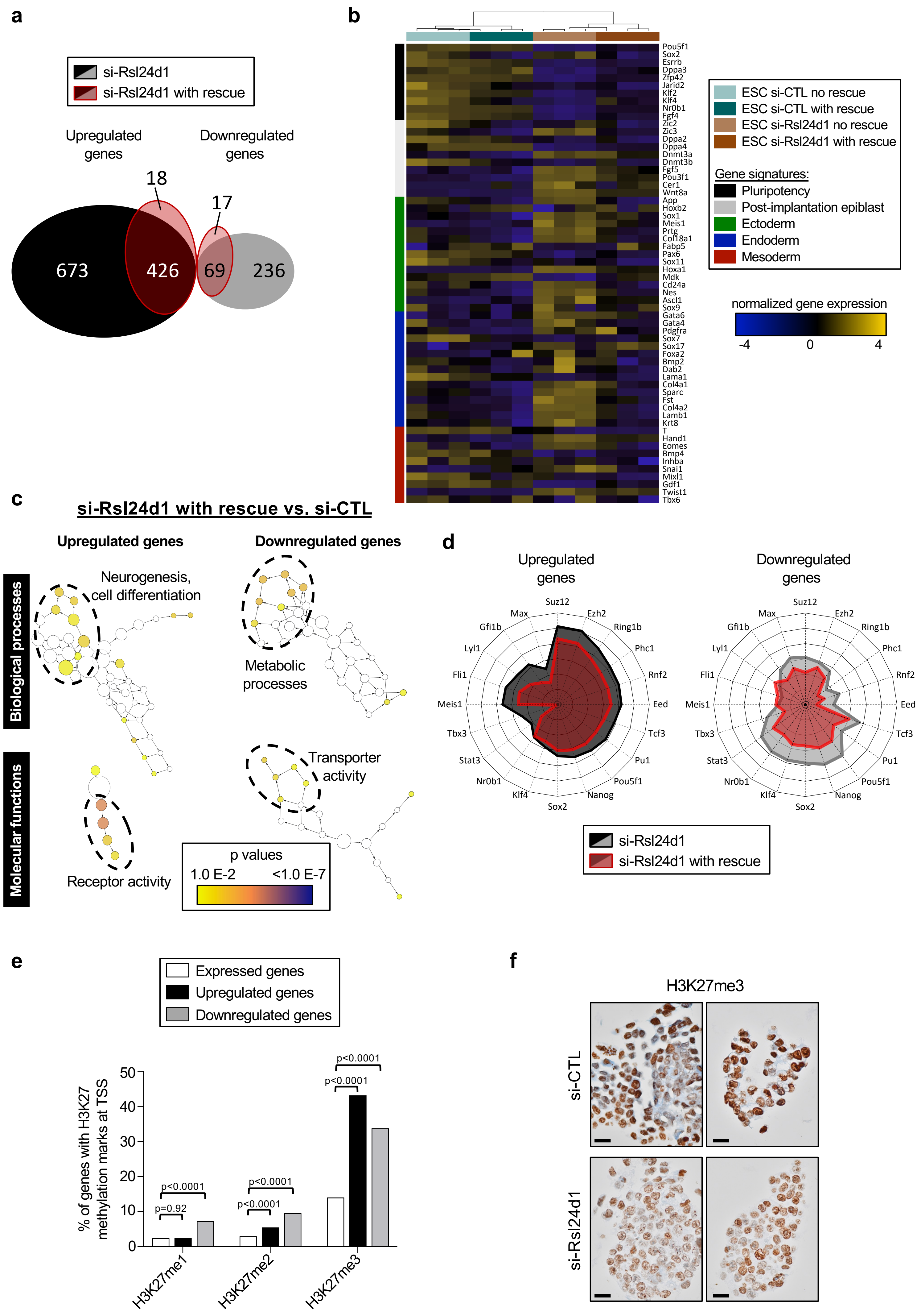
a
si-Rsl24d1
si-Rsl24d1 with rescue
Upregulated genes
Downregulated genes
18
17
673
426
69
236
b
ESC si-CTL no rescue
ESC si-CTL with rescue
ESC si-Rsl24d1 no rescue
ESC si-Rsl24d1 with rescue
Gene signatures:
Pluripotency
Post-implantation epiblast
Ectoderm
Endoderm
Mesoderm
normalized gene expression
-4
0
4
Pou5f1
Sox2
Esrrb
Dppa3
Zfp42
Jarid2
Klf2
Klf4
Nr0b1
Fgf4
Zic2
Zic3
Dppa2
Dppa4
Dnmt3a
Dnmt3b
Fgf5
Pou3f1
Cer1
Wnt8a
App
Hoxb2
Sox1
Meis1
Prtg
Col18a1
Fabp5
Pax6
Sox11
Hoxa1
Mdk
Cd24a
Nes
Ascl1
Sox9
Gata6
Gata4
Pdgfra
Sox7
Sox17
Foxa2
Bmp2
Dab2
Lama1
Col4a1
Sparc
Fst
Col4a2
Lamb1
Krt8
T
Hand1
Eomes
Bmp4
Inhba
Snai1
Mixl1
Gdf1
Twist1
Tbx6
c
si-Rsl24d1 with rescue vs. si-CTL
Upregulated genes
Downregulated genes
Biological processes
Molecular functions
Neurogenesis, cell differentiation
Metabolic processes
Transporter activity
Receptor activity
p values
1.0 E-2
<1.0 E-7
d
Upregulated genes
Downregulated genes
Suz12
Ezh2
Ring1b
Phc1
Rnf2
Eed
Tcf3
Pu1
Pou5f1
Nanog
Sox2
Klf4
Nr0b1
Stat3
Tbx3
Meis1
Fli1
Lyl1
Gfi1b
Max
si-Rsl24d1
si-Rsl24d1 with rescue
e
Expressed genes
Upregulated genes
Downregulated genes
% of genes with H3K27 methylation marks at TSS
0
10
20
30
40
50
p=0.92
p<0.0001
p<0.0001
p<0.0001
p<0.0001
p<0.0001
H3K27me1
H3K27me2
H3K27me3
f
H3K27me3
si-CTL
si-Rsl24d1

Supplemental Figure 4. RSL24D1 depletion impairs specific molecular pathways. a Venn diagram describing the overlap between differentially expressed genes in si-Rsl24d1 and si-Rsl24d1-rescue cells compared to si-CTL treated cells. **b** Gene expression heatmap for a panel of 60 markers [43] of pluripotency, post-implantation epiblast and the three germ-layers measured by RNA-seq in si-CTL, si-CTL with RSL24D1 rescue, si-Rsl24d1 and si-Rsl24d1 with RSL24D1 rescue conditions (n=3). Expression is normalized as TPM. **c** Gene ontology enrichment analyses of molecular function and biological process annotations for genes displaying significant expression changes by RNA-seq analysis between si-Rsl24d1 treated CGR8 cells expressing the ectopic RSL24D1 protein compared to si-CTL treated ESCs. The left and right panels represent hierarchical trees of the most enriched terms in genes upregulated and downregulated, respectively. The size of the nodes represents the numbers of genes associated to each GO term, and the corresponding p values defined by a hypergeometric test with Benjamini and Hochberg false discovery rate corrections are indicated by colour codes, according to the scale provided. The most represented GO term categories are indicated. The corresponding data are available in Supplementary Data 5C and 5D. **d** Radar plot summarizing the StemChecker scores for PTFs and chromatin-associated factors with a significant association score to the differentially regulated genes either in Rsl24d1-depleted or in Rsl24d1-rescue cells compared to si-CTL treated ESCs. The corresponding data are available in Supplementary Data 6. **e** Analysis of the proportion of transcription start sites (TSS) associated with H3K27 mono, bi or tri-methylation marks [7] for genes differentially expressed in Rsl24d1-depleted CGR8 cells (upregulated n=1099, black; downregulated n=305, grey) and in the gene set associated with significant expression predictions (DEseq2 corrected p values < 0.01, n=6690, white). Fisher exact test. **f** Representative images of immunohistochemistry assays stained with anti-trimethylated form of H3K27 (H3K27me3) antibodies, in ESC^{FBS} CGR8 and treated with si-CTL or si-Rsl24d1 (n=2). The scale bars represent 20 µm.

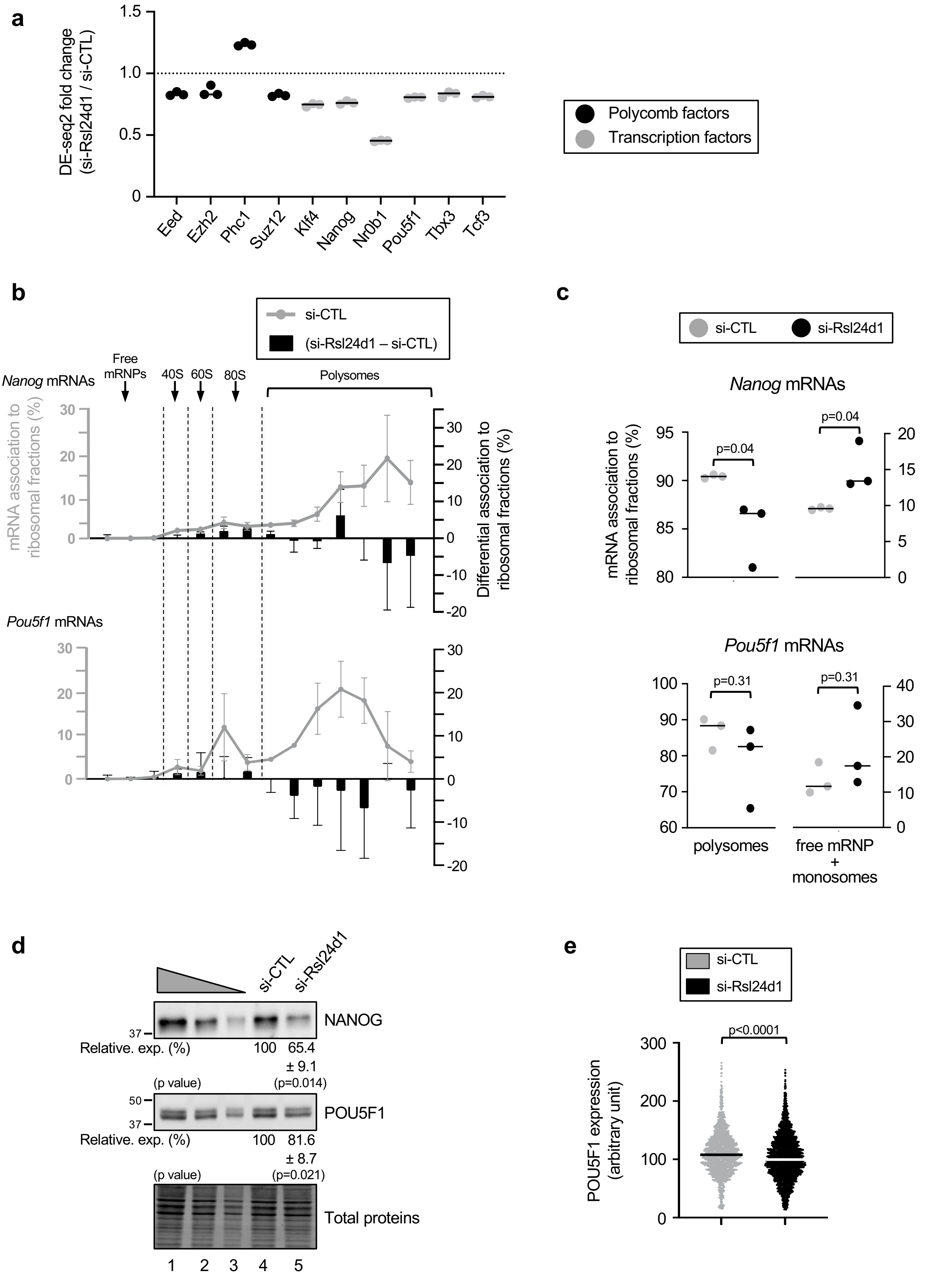

Supplemental Figure 5. Rsl24d1 downregulation alters the translation of key pluripotency factors in CGR8 cells. a Dot plots representing expression ratios estimated by DE-seq2 in si-Rsl24d1 over si-CTL treated CGR8 cells, for PRC factors (black) and PTFs (gray) analyzed in Fig. 4c. The bar represents the median (n=3). **b** Graphs representing *Nanog* and *Pou5f1* mRNA levels measured by RT-qPCR in each fraction collected from polysome profilings (gray curves, left scales) after normalization with a Luciferase spike-in mRNA and relative to the total amount of mRNAs detected in each polysome profiling (sum of all fractions) (n=3). Data are presented as mean values +/- SEM. The bar graph

represents the differential detection of each mRNA in each fraction in CGR8 cells treated with si-Rsl24d1 and si-CTL (black bars, right scales). The positions of the 40S, 60S, 80S monosomes and polysomes are indicated. **c** Dot plots representing the relative proportions of Nanog and Pou5f1 mRNAs detected by qRT-PCR in free mRNPs/monosomal or polysomal fractions, based on quantifications detailed in the supplemental Fig. 5b, in si-CTL (grey) or si-Rsl24d1 (black) treated CGR8 ESC^{FBS} (n=3). The bar represents the mean value. Unpaired two tailed Student's *t* test. **d** Representative immunoblots of NANOG (n=3) and POU5F1 (n=3) proteins in si-CTL or si-Rsl24d1 treated CGR8 ESC^{FBS}. Lanes 1 to 3 correspond to serial dilutions of si-CTL-treated ESC^{FBS} (1:1, 1:3 and 1:9, respectively). TCE-labeled total proteins are used for normalization. Quantifications of immunoblot signals normalized to total proteins and relative to the si-CTL-treated conditions (Rel. exp. (%)) are indicated. Paired two tailed Student's *t* test. **e** Quantifications of immunostaining signals from siRNA treated naive CGR8 cells stained with anti-POU5F1 antibodies and imaged by the Operetta CLS high-content analysis microscope (si-CTL, grey, n=1620; si-Rsl24d1, black, n=2740). Mean values are indicated by a black (si-CTL) and a white (si-Rsl24d1) line in the scatter plots. Unpaired two tailed Student's *t* test.

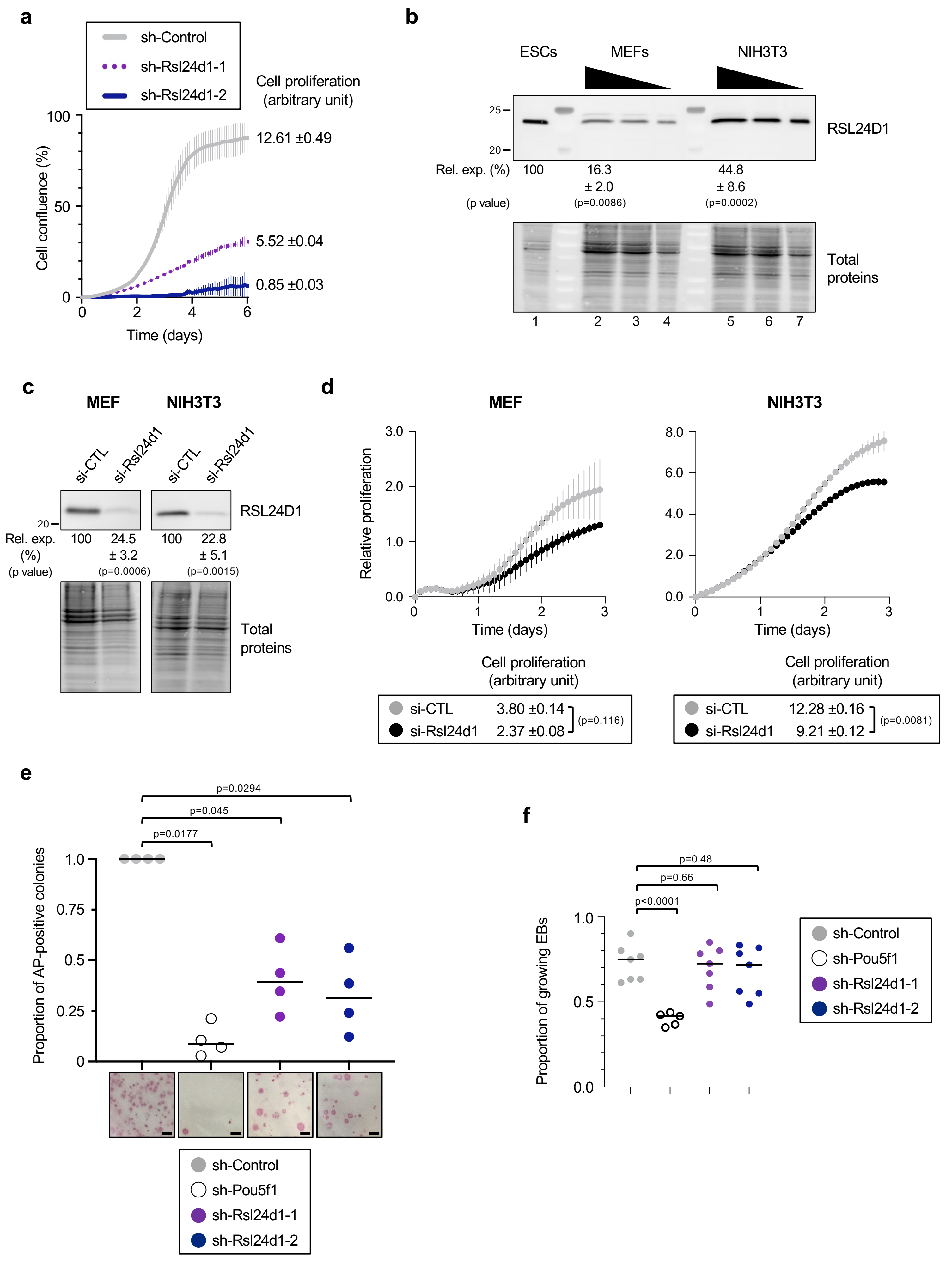
a
sh-Control
sh-Rsl24d1-1
sh-Rsl24d1-2
Cell proliferation (arbitrary unit)
12.61 ±0.49
5.52 ±0.04
0.85 ±0.03
Cell confluence (%)
100
50
0
0 2 4 6
Time (days)
b
ESCs
MEFs
NIH3T3
25
20
RSL24D1
Rel. exp. (%)
100
16.3 ± 2.0
44.8 ± 8.6
(p value)
(p=0.0086)
(p=0.0002)
Total proteins
1 2 3 4 5 6 7
c
MEF
NIH3T3
si-CTL
si-Rsl24d1
si-CTL
si-Rsl24d1
20
RSL24D1
Rel. exp. (%)
(p value)
100
24.5 ± 3.2
(p=0.0006)
100
22.8 ± 5.1
(p=0.0015)
Total proteins
d
MEF
NIH3T3
Relative proliferation
3.0
2.0
1.0
0.0
0 1 2 3
Time (days)
8.0
6.0
4.0
2.0
0.0
0 1 2 3
Time (days)
Cell proliferation (arbitrary unit)
si-CTL 3.80 ±0.14
si-Rsl24d1 2.37 ±0.08
(p=0.116)
Cell proliferation (arbitrary unit)
si-CTL 12.28 ±0.16
si-Rsl24d1 9.21 ±0.12
(p=0.0081)
e
p=0.0294
p=0.045
p=0.0177
Proportion of AP-positive colonies
1.0
0.75
0.5
0.25
0
sh-Control
sh-Pou5f1
sh-Rsl24d1-1
sh-Rsl24d1-2
f
p=0.48
p=0.66
p<0.0001
Proportion of growing EBs
1.0
0.5
0.0
sh-Control
sh-Pou5f1
sh-Rsl24d1-1
sh-Rsl24d1-2

Supplemental Figure 6. RSL24D1 expression is required to sustain ESC self-renewal. **a** Analysis of the proliferative capacities, defined as in Fig. 6a (Incucyte® technology), of CGR8 ESC^{FBS} expressing sh-Control (grey curve), sh-Rsl24d1-1 (purple curve) or sh-Rsl24d1-2 (blue curve) shRNAs (n=6). Data are presented as mean values +/- SEM. **b** Representative immunoblot of RSL24D1 expression in ESC^{FBS} (lane 1) and in two non-pluripotent mouse MEFs (lanes 2 to 4) and NIH3T3 cells (lanes 5 to 7)(n=3). Lanes 2 to 4 and lanes 5 to 7 correspond to serial dilutions of MEF and NIH3T3 total extracts (1:1, 1:3 and 1:9), respectively. TCE labeled proteins (Total Proteins) are used for normalization. Quantifications of immunoblot signals normalized to total proteins and relative to the ESC^{FBS} sample (Rel. exp. (%)) are indicated. Paired two tailed Student's *t* test. **c** Representative immunoblots of RSL24D1 expression in MEF (left panel) and in NIH3T3 (right panel) treated with si-CTL or si-Rsl24d1 (n=3). The corresponding quantifications of RSL24D1 signals normalized to total proteins and to the si-CTL condition are indicated. Paired two tailed Student's *t* test. **d** Proliferation estimated by cell confluence analysis of si-CTL and si-Rsl24d1 treated MEFs (n=3, left panel) and NIH3T3 (n=3, right panel), as previously described in a. Data are presented as mean values +/- SEM. Paired two tailed Student's *t* test. **e** Analysis of the self-renewing capacities of CGR8 ESC^{FBS} estimated by the quantification of the number of individual colonies with alkaline phosphatase activity detected by colorimetric labelling. Colonies expressing control- (n=4), Pou5f1- (n=4) or Rsl24d1-targeting shRNAs (sh-Rsl24d1-1, sh-Rsl24d1-2) (n=5) were tested. Representative images of the ESC colonies are shown. Unpaired two tailed Student's *t* test. The bar scale represents 500 µm. **f** Analysis of the proportion of 12-day old EBs generated from CGR8 cells expressing control- (n=7), Pou5f1- (n=5) or Rsl24d1-shRNAs (n=7). Unpaired two tailed Student's *t* test Student's *t* test.

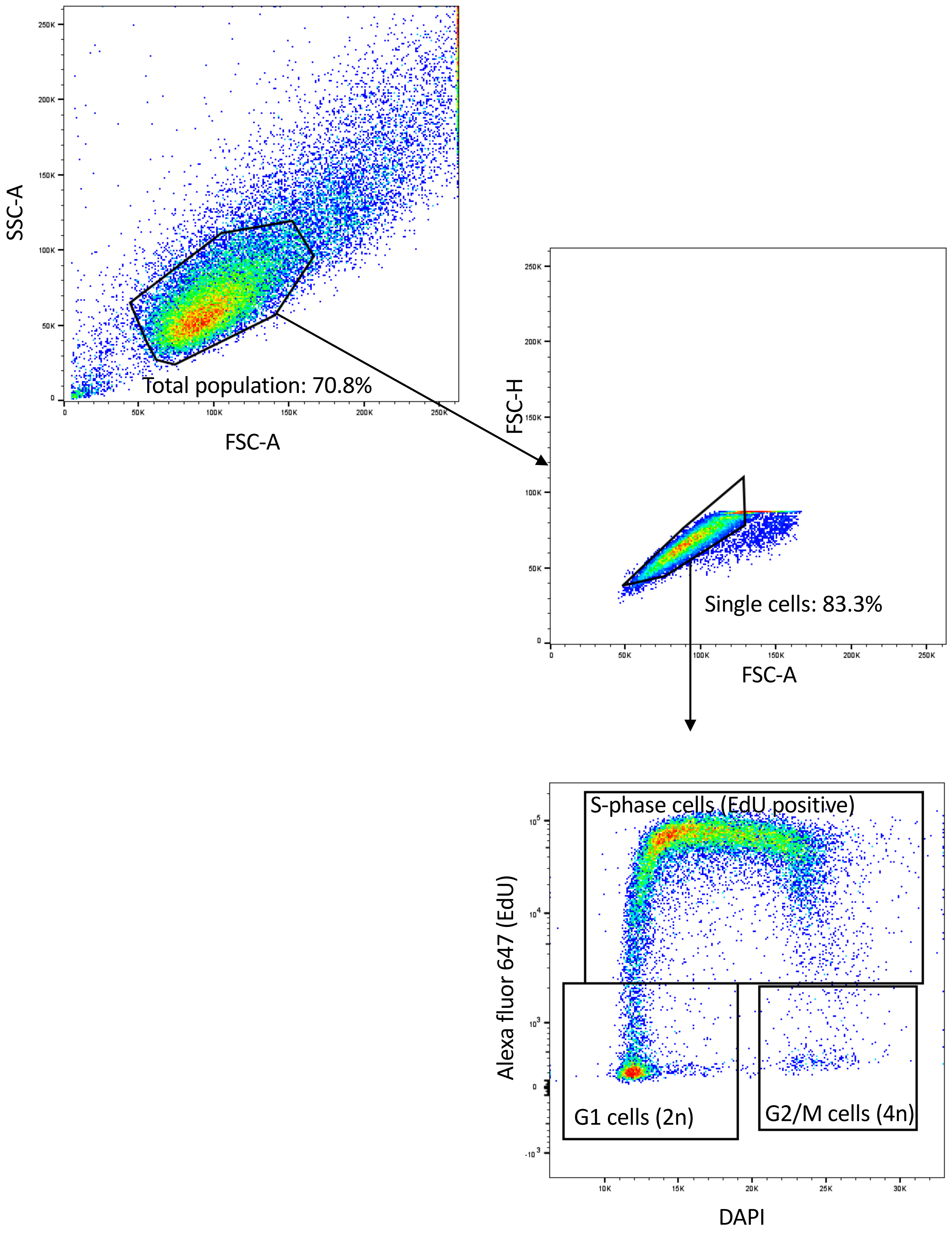

Supplemental Figure 7. Gating strategy used to define the impact of Rsl24d1 depletion on cell cycle progression in mouse ESCs. After excluding cellular debris and cell doublets, single cells were analysed based on their DNA content (2n to 4n) (FxViolet staining – DAPI channel) and based on the detection of incorporated EdU in the genomic DNA (Alexa Fluor 647 channel) to assess their distribution in the different phases of the cell cycle, as previously described [40].

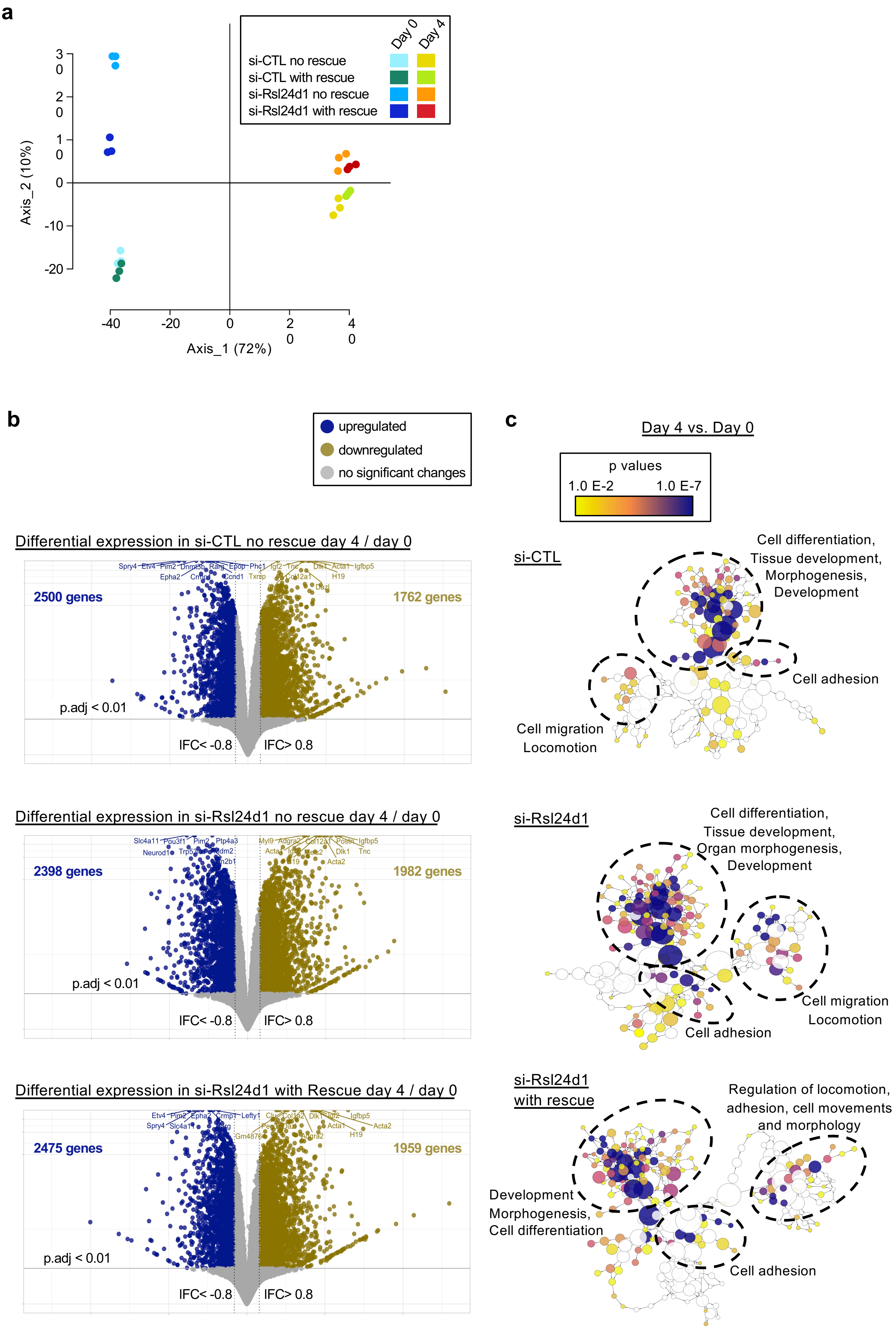
a
Day 0
Day 4
si-CTL no rescue
si-CTL with rescue
si-Rsl24d1 no rescue
si-Rsl24d1 with rescue
Axis_2 (10%)
Axis_1 (72%)
b
upregulated
downregulated
no significant changes
Differential expression in si-CTL no rescue day 4 / day 0
2500 genes
1762 genes
p.adj < 0.01
lFC< -0.8
lFC> 0.8
Differential expression in si-Rsl24d1 no rescue day 4 / day 0
2398 genes
1982 genes
p.adj < 0.01
lFC< -0.8
lFC> 0.8
Differential expression in si-Rsl24d1 with Rescue day 4 / day 0
2475 genes
1959 genes
p.adj < 0.01
lFC< -0.8
lFC> 0.8
c
Day 4 vs. Day 0
p values
1.0 E-2
1.0 E-7
si-CTL
Cell differentiation, Tissue development, Morphogenesis, Development
Cell adhesion
Cell migration Locomotion
si-Rsl24d1
Cell differentiation, Tissue development, Organ morphogenesis, Development
Cell migration Locomotion
Cell adhesion
si-Rsl24d1 with rescue
Regulation of locomotion, adhesion, cell movements and morphology
Development Morphogenesis, Cell differentiation
Cell adhesion

Supplemental Figure 8. Rsl24d1 downregulation impacts gene expression signature in CGR8 pluripotent and differentiating cells. a Principal component analysis plot of all samples analyzed by DE-seq2 at days 0 and 4. Each biological replicate (n=3) is indicated by an individual dot and the main axis are shown. **b** Volcano plots representing the differentially expressed genes between day 4 of RA treatment and day 0, respectively for si-CTL, si-RSL24d1 and si-Rsl24d1 rescue cells. Genes presenting a p value > 0.01 and a log2 fold change < 0.8 were labeled as "no significant change" (grey colour) and the number of differentially expressed genes is indicated. **c** Gene ontology enrichment analyses of biological process annotations for genes showing significant upregulation at day 4 of RA treatment in si-CTL, si-Rsl24d1 and si-Rsl24d1 with rescue CGR8 cells. Data related to panel b (brown-labeled genes). The size of the nodes represents the numbers of genes associated to each GO term, and the corresponding p values defined by a hypergeometric test with Benjamini and Hochberg false discovery rate corrections are indicated by colour codes, according to the scale provided. The most represented GO term categories are indicated. The corresponding data are available in Supplementary Data 8A-C.
