# Supplementary material for: RSL24D1 sustains steady-state ribosome biogenesis and pluripotency translational programs in embryonic stem cells
Source: Nat Commun. 2023 Jan 23;14:356. doi: 10.1038/s41467-023-36037-7 (PMC9870888; doi:10.1038/s41467-023-36037-7)
Supplement: Supplementary file 3 — Description of Additional Supplementary Files [file 41467_2023_36037_MOESM3_ESM.pdf]

Description of Additional Supplementary Files

File Name: Supplementary Data 1
Description: RNAseq-based gene expression predictions for RBFs and RPs in mouse samples.

File Name: Supplementary Data 2
Description: Mass spectrometry analysis of total extracts from si-CTL and si-Rsl24d1 treated CGR8 cells.

File Name: Supplementary Data 3
Description: Gene ontology enrichment analysis of up- and downregulated proteins in RSL24D1 depleted ESCs.

File Name: Supplementary Data 4
Description: DEseq2 analysis of si-CTL, si-Rsl24d1 no rescue, siCTL with rescue and si-Rsl24d1 with rescue at day 0 and day 4 of differentiation.

File Name: Supplementary Data 5
Description: GO annotation enrichment analysis of genes differentially expressed in Rsl24d1-depleted undifferentiated ESCs (day 0).

File Name: Supplementary Data 6
Description: Stemchecker analysis of differentially expressed genes in Rsl24d1-depleted ESCs.

File Name: Supplementary Data 7
Description: Analysis of transcription and chromatin factor binding sites.

File Name: Supplementary Data 8
Description: GO annotation enrichment analysis of genes upregulated upon ESC differentiation.

File Name: Supplementary Data 9
Description: Supplementary method information (primers, shRNAs, siRNAs and antibodies).
