# Supplementary material for: RSL24D1 sustains steady-state ribosome biogenesis and pluripotency translational programs in embryonic stem cells
Source: Nat Commun. 2023 Jan 23;14:356. doi: 10.1038/s41467-023-36037-7 (PMC9870888; doi:10.1038/s41467-023-36037-7)
Supplement: Supplementary file 13 — Reporting Summary [file 41467_2023_36037_MOESM13_ESM.pdf]

nature portfolio

Corresponding author(s): Mathieu GABUT

Last updated by author(s): Jan 7, 2023

Reporting Summary

Nature Portfolio wishes to improve the reproducibility of the work that we publish. This form provides structure for consistency and transparency in reporting. For further information on Nature Portfolio policies, see our Editorial Policies and the Editorial Policy Checklist.

Statistics

For all statistical analyses, confirm that the following items are present in the figure legend, table legend, main text, or Methods section.

n/a	Confirmed	
☐	☒	The exact sample size (*n*) for each experimental group/condition, given as a discrete number and unit of measurement
☐	☒	A statement on whether measurements were taken from distinct samples or whether the same sample was measured repeatedly
☐	☒	The statistical test(s) used AND whether they are one- or two-sided<br>*Only common tests should be described solely by name; describe more complex techniques in the Methods section.*
☐	☒	A description of all covariates tested
☒	☐	A description of any assumptions or corrections, such as tests of normality and adjustment for multiple comparisons
☐	☒	A full description of the statistical parameters including central tendency (e.g. means) or other basic estimates (e.g. regression coefficient) AND variation (e.g. standard deviation) or associated estimates of uncertainty (e.g. confidence intervals)
☐	☒	For null hypothesis testing, the test statistic (e.g. *F*, *t*, *r*) with confidence intervals, effect sizes, degrees of freedom and *P* value noted<br>*Give P values as exact values whenever suitable.*
☒	☐	For Bayesian analysis, information on the choice of priors and Markov chain Monte Carlo settings
☒	☐	For hierarchical and complex designs, identification of the appropriate level for tests and full reporting of outcomes
☒	☐	Estimates of effect sizes (e.g. Cohen's *d*, Pearson's *r*), indicating how they were calculated

Our web collection on statistics for biologists contains articles on many of the points above.

Software and code

Policy information about availability of computer code

Data collection	The data collection is described in the Methods section. No custom software was used.
Data analysis	The data analysis is described in the Methods section. The AP colonies were quantified using the Image J 1.52a software and Java 1.8.0. The cell proliferation analysis was performed with the Incucyte Software v2020c. The cell cycle analysis was performed with FlowJo software v10.8. The western blots were quantified using the ImageLab software (Bio-Rad) 6.0.1. For the proteomics data, proteins were identified using MaxQuant (1.6.17.0) and the corresponding statistical analyses were achieved with the ProStaR 1.28.0 software. The images obtained from the Operetta HCS system were analysed with the Harmony 4.8 and the Columbus softwares (Perkin Elmer). Protein sequence comparisons were performed with the online Clustal Omega software and visualized using the Jalview 2.11.0 software. The protein structures were predicted using the on line tool Swiss-Model structures assessment tool (https://swissmodel.expasy.org/assess) and Pymol 2.3.5 software. The RNA-seq data were analysed with FastQC (0.11.5), STAR (2.7.0f), RSeQC (3.0.0), Salmon (0.14.1), R (3.6.1), Tximport (v1.12.3) 72 and DESeq2 (v1.24). Gene Ontology analyses were performed with BiNGO (v3.0.3) and Cytoscape (v3.7.2). Stemness signatures were estimated with the online tool Stemchecker (http://stemchecker.sysbiolab.eu/). Graphs and statistical analyses were otherwise performed with the PRISM 9 and excel 16.63.1 softwares. Figures were generated with microsoft powerPoint 16.63.1.

For manuscripts utilizing custom algorithms or software that are central to the research but not yet described in published literature, software must be made available to editors and reviewers. We strongly encourage code deposition in a community repository (e.g. GitHub). See the Nature Portfolio guidelines for submitting code & software for further information.

Data

Policy information about availability of data

All manuscripts must include a data availability statement. This statement should provide the following information, where applicable:

- Accession codes, unique identifiers, or web links for publicly available datasets
- A description of any restrictions on data availability
- For clinical datasets or third party data, please ensure that the statement adheres to our policy

The publicly available dataset used in this study can be accessed under the GEO accession codes GSE45505, GSE89211, GSE116603 and GSE85717. The ChIP-seq data can be accessed in the ChIP-Atlas database (https://chip-atlas.org/). The protein structures are available in the Protein DataBank (https://www.rcsb.org/) under the accessions PDB-6N8J, PDB-6LSS. The RNA-seq data profiles generated in this study have been deposited to NCBI GEO (GSE218290) and is publicly available. The mass spectrometry proteomics data have been deposited to the ProteomeXchange Consortium via the PRIDE 75 partner repository with the dataset identifier PXD030497. All other data supporting the findings of the study are available in this article, its supplementary information files or from the corresponding author upon reasonable request.

Human research participants

Policy information about studies involving human research participants and Sex and Gender in Research.

Reporting on sex and gender	not applicable
Population characteristics	not applicable
Recruitment	not applicable
Ethics oversight	not applicable

Note that full information on the approval of the study protocol must also be provided in the manuscript.

Field-specific reporting

Please select the one below that is the best fit for your research. If you are not sure, read the appropriate sections before making your selection.

☒ Life sciences ☐ Behavioural & social sciences ☐ Ecological, evolutionary & environmental sciences

For a reference copy of the document with all sections, see nature.com/documents/nr-reporting-summary-flat.pdf

Life sciences study design

All studies must disclose on these points even when the disclosure is negative.

Sample size	No sample size calculations were performed. Unless otherwise stated, all experiments included at least independent repeats in triplicates. Sample size was determined based on standards for experimental cell biology studies, attempting to have a minimum of N = 3 biological replicates with sufficient reproducibility. For some immunostaining assay in Suppl. fig 5e, the experiments were done twice, but we counted several thousands of cells to quantify biological changes in a reliable manner. For the RNAseq analyses, a primary component analysis was performed to establish sample-to-sample variability between biological replicates and the different conditions tested.
Data exclusions	No data were excluded from the analyses conducted in this manuscript.
Replication	All experiments were repeated at 3 times or more using the same conditions. All experiments were quantified individually and we reported the average values observed together with standard deviations. No data or experiment was preferentially excluded unless a major technical issue was revealed by the positive control metrics, attempts of data replication were, therefore, successful from a technical point of view.
Randomization	This study did not rely on data randomization as all data were experimentally defined either by the authors for all the de novo data, or by other experimentators for previously published data used in this study.
Blinding	Blinding was not relevant to this study as all conditions or treatments were compared to control groups defined by the experimentators for direct comparisons. This study did not rely on randomized dataset.

Reporting for specific materials, systems and methods

We require information from authors about some types of materials, experimental systems and methods used in many studies. Here, indicate whether each material, system or method listed is relevant to your study. If you are not sure if a list item applies to your research, read the appropriate section before selecting a response.

Materials & experimental systems

n/a	Involved in the study	
☐	☒	Antibodies
☐	☒	Eukaryotic cell lines
☒	☐	Palaeontology and archaeology
☒	☐	Animals and other organisms
☒	☐	Clinical data
☒	☐	Dual use research of concern

Methods

n/a	Involved in the study	
☒	☐	ChIP-seq
☐	☒	Flow cytometry
☒	☐	MRI-based neuroimaging

Antibodies

Antibodies used

All antibodies used in this study and the conditions are detailed in the Supplemental Table S9B.

Validation

All antibodies used in this study are commercially available and selected according to the manufacturer's provided results. For the RSL24D1 antibody which has been extensively used in the manuscript, its specificity has been first validated in mouse cells (ESC, NIH3T3 and MEFs) by western blot and immuno-fluorescence assays, using siRNAs or shRNA depletion assays or RSL24D1 overexpression conditions. The specificity of RSL24D1 antibody was only assessed by western blot in human tissue and cell samples based on protein sequence conservation (>98% conservation between mouse and human) and conserved migration pattern (band detected at the same molecular weight in mouse and human cellular extracts).
- RPL24 antibody (Invitrogen, PA5-30157) recognize the mouse and human RPL24 proteins. Its specificity was assessed by knock down in Hela cells by western blot.
- The anti-RPL8 antibody recognizes the endogenous RPL8 human protein (28 KDa) and is predicted to react with the mouse and rat proteins. It has been tested by western blot and immunoprecipitation assays. We confirmed it also detects a 28 KDa band in mouse protein extracts.
- The S6 Ribosomal Protein (5G10) Rabbit Monoclonal Antibody detects endogenous levels of total S6 ribosomal protein independent of phosphorylation. It reacts with the human, mouse and rat RPS6 proteins and was tested by western blot on human, mouse and monkey cell extracts, as well as by immunofluorescence (IF) on mouse brain tissues and human cancer samples.
- The eIF6 (D16E9) XP® Rabbit Monoclonal Antibody detects endogenous levels of eIF6 in mouse and human cell lines by western blot and was also tested for IF assays in Hela cells.
- The polyclonal GAPDH antibody was raised against a synthetic peptide of the mouse GAPDH protein and was succesfully used for western blot and IF assays in several publications.
- The anti-Histone H3 antibody (ab1791) reacts with Histone H3 from numerous species including Mouse, Rat and Human and has been used in over 3800 publications, including for ChIP-seq assays.
- The anti-FBL antibody (Genetex, GTX101807) reacts with the mouse, rat and Human proteins. It detexts a 38 KDa band on SDS-PAGE gels by western blot and has been reported in 2 publications for immunoprecipitation assays.
- the anti-POU5F1 antibody (BD Biosciences, 611203) was succesfully used on human and mouse ESC whole cell extracts for western blot and IF assays. It has been cited in 6 references.
- the anti-mouse NANOG antibody has been derived against a peptide of the mouse Nanog protein and tested for western blot, flow cytometry and IF assays. It has been referenced in 20 publications.
- the Anti-EED Antibody, clone AA19 is a mouse monoclonal antibody for detection of EED also known as WD protein associating with embryonic ectoderm development and has been validated in WB. It has been referenced in 8 publications.
- the Ezh2 (D2C9) XP® Rabbit mAb detects endogenous levels of total Ezh2 protein. This antibody does not cross-react with Ezh1 protein and reacts with the Human, Mouse, Rat, Monkey EZH2 protein. It has been widely used for western blot, IF, IHC an dIP assays in over 600 publications.
- the SUZ12 (D39F6) XP® Rabbit mAb detects endogenous levels of SUZ12 protein from Human, Mouse, Rat and Monkey. It has been extesnively used for western blot, flow cytometry and chromatin-IP assays, and it has been cited in over 200 publications.
- the Anti-β-Actin antibody was raised against the N-terminal peptide of the protein. It has been reported for western blot and IF assays in several publications.
- The anti-H3K27me3 ChIP grade Antibody reacts with the H3K27me3 protein from Human, mouse, rat, pig, zebrafish, Drosophila, Schistosoma, Arabidopsis and cow. It has been used for Dot blot, IF, ChIP-seq and western blot assays.

Eukaryotic cell lines

Policy information about cell lines and Sex and Gender in Research

Cell line source(s)

Mouse CGR8 ESCs were obtained from the ECACC General Collection. R1 and G4 cells were kindly gifted from Andras Nagy (University of Toronto, Canada). CD-1 MEFs were obtained from Stemcell technologies. NIH3T3 (ATCC, CRL-1658) and HEK-293 (ECACC, 93061524) cells were obtained from ATCC and ECACC General Collections.

Authentication

None of the cell line used were authenticated.

Mycoplasma contamination

All cell lines were routinely tested for mycoplasma contaminations and tested negative.

Commonly misidentified lines
(See ICLAC register)

no commonly misidentified lines were used in this study.

Flow Cytometry

Plots

Confirm that:

☒ The axis labels state the marker and fluorochrome used (e.g. CD4-FITC).

☒ The axis scales are clearly visible. Include numbers along axes only for bottom left plot of group (a 'group' is an analysis of identical markers).

☒ All plots are contour plots with outliers or pseudocolor plots.

☒ A numerical value for number of cells or percentage (with statistics) is provided.

Methodology

Sample preparation	CGR8 cells were cultured on gelatin coated cell culture dishes and incubated with 10 uM EdU prior to fixation to assess the cell cycle phases distribution.
Instrument	LSR Fortessa Cytometer
Software	FlowJo software
Cell population abundance	Cell populations range from 2% to 80%. Cell population purity was assessed based on EdU incorporation levels and nuclear genomic DNA content.
Gating strategy	First, we defined gating based on the SSC/FCS channels to remove remained cell doublets, debris, and contaminating particulates. Next, manual gating was performed on DAPI channel (FxViolet), and Alexa Fluor 647 (Click-iT EdU Alexa Fluo 647) to identify cell-cycle stages, based on untreated control cells (No Edu treatment, No Fx Violet staining).

☒ Tick this box to confirm that a figure exemplifying the gating strategy is provided in the Supplementary Information.
